# Supplementary material for: Drosophila p53 isoforms have overlapping and distinct functions in germline genome integrity and oocyte quality control
Source: eLife. 2022 Jan 13;11:e61389. doi: 10.7554/eLife.61389 (PMC8758136; doi:10.7554/eLife.61389)
Supplement: Supplementary file 1. [file elife-61389-supp1.docx]

| **Control** | **Mutant Strain** | **S1 N.C.^2^** | **S1 O^3^** | **S2 N.C.^2^** | **S2 O^3^** | **S3 N.C.^2^** | **S3 O^3^** | **S4 N.C.^2^** | **S4 O^3^** |
| --- | --- | --- | --- | --- | --- | --- | --- | --- | --- |
| Wild Type | p53A-B- | 0.0163 | 0.0193 | 0.02 | 0.02 | 0.17 | 0.17 | 0.04 | 0.04 |
| Wild Type | p53A-B+ | 0.42 | 0.05 | 1 | 1 | 1 | 1 | 1 | 1 |
| Wild Type | p53A+B- | 0.0001 | 0.0001 | N.D.^3^ | 0.0011 | 1 | 1 | 1 | 1 |
| Wild Type | okra^-^; p53A+B+ | 0.1192 | 0.0002 | 0.154 | 0.0013 | 0.05 | 0.06 | N.D | N.D. |
| Wild Type | okra^-^; p53A-B- | 0.0071 | 0.0028 | 0.0003 | N.D | 0.0002 | 0.0008 | 0.0138 | 0.0005 |
| Wild Type | okra^-^; p53A-B+ | N.D. | 0.0001 | N.D. | N.D | N.D. | 0.0028 | N.D. | 0.0121 |
| Wild Type | okra^-^; p53A+B- | N.D. | 0.0001 | N.D. | N.D | N.D. | N.D. | N.D. | N.D. |
| okra^-^ | okra^-^; p53A-B- | 0.9579 | 0.0054 | 0.0299 | 0.1295 | 0.0002 | 0.0185 | 0.0026 | 0.0001 |
| okra^-^ | okra^-^; p53A-B+ | 0.7381 | 0.2366 | 0.154 | 0.2366 | 0.05 | 0.06 | N.D | 0.0012 |
| okra^-^ | okra^-^; p53A+B- | 0.1959 | 0.2366 | 0.0653 | 0.2366 | 0.0005 | 0.0411 | N.D. | N.D. |

Supplemental File 1. p values for frequency of nurse cells and oocytes with DNA breaks^1^

1: Related to Figures 6 and 7, p values calculated using unpaired student's t test

2 N.C: Nurse cells

3: N.D.: Not Determined since SEM is zero
