# Supplementary material for: Drosophila p53 isoforms have overlapping and distinct functions in germline genome integrity and oocyte quality control
Source: eLife. 2022 Jan 13;11:e61389. doi: 10.7554/eLife.61389 (PMC8758136; doi:10.7554/eLife.61389)
Supplement: Supplementary file 2. [file elife-61389-supp2.docx]

| **Control** | **Compared to** | **Oocyte** | **rank on plot** | **Nurse Cell** | **rank on plot** |
| --- | --- | --- | --- | --- | --- |
| Wildtype | p53A-B- | <0.0001 | **** | <0.0001 | **** |
| Wildtype | p53A-B+ | 0.0047 | ** | 0.0104 | * |
| Wildtype | p53A+B- | <0.0001 | **** | <0.0001 | **** |
| Wildtype | okra- | <0.0001 | **** | <0.0001 | **** |
| Wildtype | okra-; p53A-B- | <0.0001 | **** | <0.0001 | **** |
| Wildtype | okra-; p53A-B+ | <0.0001 | **** | x0.9995 | ns |
| Wildtype | okra-; p53A+B- | <0.0001 | **** | <0.0001 | **** |
| p53A-B- | okra-; p53A-B- | 0.1239 | ns | 0.9102 | ns |
| p53A-B+ | okra-; p53A-B+ | <0.0001 | **** | 0.555 | ns |
| p53A+B- | okra-; p53A+B- | <0.0001 | **** | <0.0001 | **** |
| okra- | okra-; p53A-B- | 0.0026 | ** | 0.795 | ns |
| okra- | okra-; p53A-B+ | 0.1334 | ns | <0.0001 | **** |
| okra- | okra-; p53A+B- | 0.009 | ** | <0.0001 | **** |

Supplementary File 2. ANOVA p value comparisons among genotypes for mean γ-H2AV intensity in stage 1 oocytes and nurse cells.^1^

1: Two-way ANOVAs were performed with pairwise adjusted p values computed through a Tukey test using GraphPad Prism (version 9.1.2). Related to Figure 6-figure supplement 2.
